# Enhancing social-emotional skills in early childhood: intervention study on the effectiveness of social and emotional learning

**DOI:** 10.1186/s40359-024-02280-w

**Published:** 2024-12-18

**Authors:** Rikuya Hosokawa, Yuki Matsumoto, Chizuko Nishida, Keiko Funato, Aki Mitani

**Affiliations:** 1 Department of Human Health Sciences, Graduate School of Medicine, Kyoto University, 53 Shogoin-kawahara-cho, Sakyo-ku, Kyoto, 606-8507 Japan; 2 School of Human Life Sciences, Tokushima Bunri University, Tokushima, 770-8514 Japan; 3 Tanabe City Shinjyo Daini Elementary School, Wakayama, 646-0011 Japan; 4 Shirahama Town Shirahama Gakudo Nursery School, Wakayama, 649-2211 Japan; 5 Minabe Ainosono Childcare Center, Wakayama, 645-0004 Japan

**Keywords:** Fun FRIENDS program, Social-emotional skills, Social and emotional learning, Kindergarten

## Abstract

**Background:**

Social and emotional learning (SEL) is crucial for developing skills such as emotional regulation, cooperation, and goal achievement. Deficits in these skills are linked to later academic and behavioral problems. While SEL interventions have been widely implemented internationally, few have been tested in early childhood settings in Japan, and their effectiveness remains unclear. In 2022, the Fun FRIENDS social and emotional learning program was introduced in class units for 4–5-year-olds attending kindergartens and children’s schools in Wakayama Prefecture, Japan. Thus, this study examined the effectiveness of the Fun FRIENDS program in children aged 4–5 years in Japan.

**Methods:**

Fun FRIENDS is based on a cognitive-behavioral approach designed to teach participants how to cope with anxiety and stress, and to develop resilience and confidence. The program consists of 10 weekly sessions of approximately 1 h each. The intervention group comprised 115 children from two facilities, while the control group comprised 93 children from three facilities. Intervention effectiveness was evaluated through changes in externalizing (e.g., aggression, oppositional behavior) and internalizing behaviors (e.g., anxiety, depression), measured using the Strengths and Difficulties Questionnaire. Finally, data from 94 participants in the intervention group and 66 participants in the control group were analyzed.

**Results:**

In the intervention group, externalizing behaviors showed a significant reduction both before and after the intervention and internalizing behaviors also decreased significantly. Conversely, the control group did not exhibit any significant changes in either externalizing or internalizing behaviors before or after the intervention. The effect size for externalizing behaviors in the target group was 0.744, while the effect size for internalizing behaviors was 0.653.

**Conclusions:**

Social and emotional learning programs in classrooms may effectively control problem behaviors in early childhood and prevent school maladjustment. A whole-class approach can reach more children, maximizing the preventive effects. The program can improve academic performance and social adaptation, contributing to the long-term development of psychological health and social skills. Nevertheless, further longitudinal research is required to assess the program’s long-term effects. In summary, strengthening the focus on social-emotional learning in early childhood education policy is key to realizing long-term benefits for child development.

**Supplementary Information:**

The online version contains supplementary material available at 10.1186/s40359-024-02280-w.

## Background

In recent years, the role of social support in children's psychological development has gained increasing attention [1]. Social support networks have been found to positively influence parenting practices and facilitate children's emotional and psychological adjustment. Early social support acts as a protective factor against developmental risks in children, such as anxiety and depression. Furthermore, parenting styles are crucial in determining children's psychological outcomes [2]. For example, authoritative and supportive parenting styles are associated with better emotional regulation and reduced internalizing and externalizing behaviors. Conversely, limited social support and disengaged parenting can exacerbate children's psychological difficulties. Taken together, these observations highlight the importance of a supportive parenting environment. Indeed, research on the long-term effects of parenting practices emphasizes the importance of consistent emotional support from parents [3].

The recognition of social support varies by gender, which also affects children's development [4]. Studies suggest that boys and girls differently perceive and respond to social support. Girls tend to be more sensitive to the relational aspects of support, while boys focus more on its practical aspects. Understanding these gender differences is vital for developing targeted support strategies.

Social-emotional skills (also called non-cognitive skills) include the ability to control emotions, cooperate with others, and achieve goals [5–7]. As children spend a lot of time in kindergarten and nursery schools before starting school, these environments play an important role in the development and improvement of social-emotional skills. The literature on these skills focuses on early childhood education and care.

Recent meta-analyses and systematic reviews have highlighted the growing recognition of the critical role social-emotional skills play in early childhood development. These skills, which include emotional regulation, social competence, and interpersonal communication, are fundamental to academic success, mental health, and overall well-being. A systematic review by Jones et al. [8] examined 35 studies on the effectiveness of social-emotional learning (SEL) programs in early childhood and found that the programs significantly improved children’s social skills, emotional regulation, and academic outcomes. Similarly, a meta-analysis by Durlak et al. [9] analyzed over 200 studies and demonstrated that SEL interventions in early childhood led to improvements in emotional distress, behavior problems, and academic performance. A more recent meta-analysis by Wilkins et al. [10] focused on the long-term effects of SEL programs; early social-emotional interventions were found to have lasting benefits, particularly in preventing mental health issues and fostering positive relationships. These studies highlight the importance of integrating social-emotional skill development into early childhood education, particularly considering the significant impact such skills have on later developmental outcomes.

SEL programs aim to equip children with the skills necessary for emotional regulation, interpersonal relationships, and problem-solving. While these programs have been globally successfully, the context in which they are implemented significantly influences their effectiveness. Notably, Japan’s unique cultural and educational setting compels careful consideration of best practices for introducing SEL programs. One prominent factor is Japan’s strong cultural emphasis on collectivism and social harmony (wa). These values encourage behaviors that prioritize group cohesion and conformity, often at the expense of individual emotional expression. Along these lines, Japanese children are typically taught to suppress outward signs of personal distress [11]. This emphasis on collectivism can both support and challenge SEL implementation. On the one hand, children are often encouraged to consider others' perspectives and cooperate, which aligns with SEL goals. On the other hand, there is a tendency to prioritize conformity over individual emotional expression, potentially limiting opportunities for children to practice self-advocacy and emotional articulation within SEL frameworks [12, 13]. Additionally, Japan's education system places significant emphasis on academic achievement, even in early childhood. The pressure to perform academically often overshadows the importance of non-academic skills, such as social-emotional competencies. Studies have shown that Japanese parents and educators may undervalue SEL compared to cognitive and academic development, posing a barrier to the integration of SEL programs [14]. Furthermore, the structured and hierarchical nature of Japanese classrooms may influence how SEL programs are delivered. Notably, teachers in Japan often adopt an authoritative role, which may contrast with the participatory and dialogic teaching methods commonly used in SEL programs [15]. Moreover, Japan’s rigid educational system, which is highly focused on academic success and standardized testing, can limit the time and resources available for SEL programs. The intense academic pressure placed on students, particularly in the highly competitive context of university entrance exams, often leaves little room for curricula that focus on emotional development and interpersonal skills [16]. SEL curricula must be adapted to align with these cultural and structural norms of the Japanese context is essential for the curricula’s successful implementation; in particular, SEL programs must resonate with local values and practices while maintaining their core objectives.

Furthermore, the integration of SEL programs into Japan’s educational system requires careful adaptation of Western frameworks to account for these cultural differences. Kawai and Kuroki [17] emphasize the importance of cultural sensitivity when designing SEL interventions, suggesting that programs must be tailored to the collective values of Japanese society to be perceived as relevant and effective. For example, SEL programs that incorporate group-based activities that foster cooperation, mutual support, and shared responsibility may be more easily accepted in Japan than those that focus on individual emotional regulation and expression [18].

Studies also highlight the potential role of social support networks in Japan, which can play a crucial role in the successful adoption of SEL programs. Japanese children often rely on family and peer networks for emotional support, and integrating these networks into SEL initiatives may enhance program effectiveness [19]. However, the challenge remains in overcoming potential resistance from educators and parents who may perceive SEL programs as a deviation from traditional educational values that emphasize academic achievement and discipline.

In Japan, the 2018 Kindergarten Education Guidelines set out 10 images that children should have acquired by the end of their early childhood years, as guidelines applicable across kindergartens, nursery schools, and certified childcare centers [20]. In addition to cognitive abilities such as “sound mind and body” and “interest in and sense of numbers, shapes, letters, etc.,” these guidelines also include concepts such as “independence (having one’s own ideas, not giving up until the end, and acting with confidence)” and “cooperativeness (communicating ideas, cooperating towards a common goal, exchanging opinions, solving problems together, and taking actions that give a sense of fulfillment).”

Furthermore, regarding the problems of first-year students (phenomena of maladjustment to school life at the time of entrance), it is necessary to provide guidance during the transition period from infancy to school age, and appropriate support for maladjustment to school (manifesting as truancy, withdrawal from society, and problematic behavior). The problem of continuity from infancy to school age is a high-risk factor for low-income households, and there is a possibility that it will lead to a cycle of poverty [21, 22]. Although the importance of preschool education support is also highlighted in the literature, approaches for effectively developing socio-emotional skills are not clearly set out. In addition, although the effectiveness of preschool education policies targeting low-income households has been confirmed overseas [23], similar policies are limited in Japan, and because low-income households are not concentrated in specific areas, it is difficult to adopt a uniform approach. Therefore, to implement effective policies in Japan, it is necessary to take a universal approach rather than targeting only high-risk households.

Broadly, SEL is a style of learning that fosters social-emotional skills. The concept was first defined by the American non-profit organization, the Collaborative for Academic, Social, and Emotional Learning (CASEL), established in 1994 [24]. The CASEL identified five competencies that children acquire through SEL, which are self-awareness, self-management, social awareness, relationship skills, and responsible decision-making. Several universal SEL programs have been developed, with reports on their effectiveness primarily originating from studies conducted overseas [25–27]. The Fun FRIENDS program is a structured SEL intervention grounded in cognitive behavioral therapy (CBT) and social learning theory, developed by Professor Paula Barrett and colleagues at the University of Queensland in Australia. Designed for a specific developmental stage (children aged 4–7 years), the aims of Fun FRIENDS are to reduce anxiety, improve coping strategies, and promote positive social interactions. Reports from Australia, the UK, the US, and Canada indicate that the program is effective in decreasing anxiety and depression, developing social skills and cognitive abilities, and improving resilience and self-esteem [28–31]. In addition, many studies have shown that preventive interventions in early childhood can improve mental health in adolescence and adulthood [32–34].

The two foundations of the Fun FRIENDS program—CBT and social learning theory—are particularly relevant to Japan's educational environment. CBT is widely recognized for its ability to help children manage anxiety, stress, and emotional regulation by teaching skills such as cognitive restructuring and problem-solving [35]. These principles are particularly beneficial for young children in Japan, where academic achievement and social conformity are often emphasized from an early age. Research shows that the pressures associated with these cultural norms can lead to heightened levels of anxiety and social withdrawal in children [36]. In step with Japan’s broader emphasis on social conformity, Japan's educational system heavily highlights group harmony and collective achievement; however, this focus can create challenges for children who struggle with social interaction or academic performance [37]. The Fun FRIENDS program, through its CBT-based structure, helps children cope with these stresses by encouraging the development of coping strategies for managing both academic and social challenges. By using techniques such as cognitive restructuring, the program assists children in identifying negative thought patterns related to school performance or peer relationships—common sources of stress in the Japanese context. Meanwhile, social learning theory, which emphasizes learning through observation and reinforcement, supports the program's focus on modeling positive behaviors. Children learn from both their peers and facilitators, reinforcing adaptive social and emotional responses to stressful situations [38]. This approach is particularly relevant in Japan, where children are often observed by their peers and teachers in highly structured settings. The Fun FRIENDS program thus offers a framework for teaching children how to navigate these challenges with confidence, encouraging them to develop the skills necessary for positive emotional regulation and effective social interaction.

While SEL programs like Fun FRIENDS have shown considerable promise in supporting children’s emotional and social development globally, their success in Japan depends on their alignment with collectivist Japanese values, such as social harmony, respect for others, and group cohesion. Along these lines, versions of Fun FRIENDS adapted to foster Japanese values of group cohesion and social harmony have been successfully implemented in Japan. More specifically, such adaptations of Fun FRIENDS to the Japanese context promote group-based activities in which children collaborate to solve problems and learn essential skills, such as expressing emotions and supporting peers. By using activities that emphasize mutual support, empathy, and cooperative learning, Fun FRIENDS helps children learn how to navigate social situations while maintaining group harmony. For instance, children are encouraged to work together in pairs or small groups and offer emotional support to one another—practices that align with the collectivist emphasis on interdependence. Additionally, role-playing exercises that encourage conflict resolution in a group context help children practice how to maintain harmony and avoid conflict, which is particularly important in Japanese culture. Furthermore, culturally relevant examples and stories are integrated into the program, demonstrating how individual actions can influence a group’s collective well-being. These adjustments help children recognize the importance of social harmony for both personal relationships and group cohesion. Along these lines, research has demonstrated that fostering collaborative behaviors and group responsibility in SEL programs enhances their impact in collectivist cultures, leading to better social outcomes for children [39, 40]. Together, these adaptations ensure that the Fun FRIENDS program aligns with the cultural values of Japan while promoting essential social-emotional skills. Ultimately, by integrating these culturally relevant elements, the program supports children in developing both individual emotional resilience and strong connections with their peers, which are essential for their long-term social and emotional well-being.

The Fun FRIENDS program consists of several key components, including emotional regulation techniques through which children learn to recognize and challenge negative thought patterns and replace them with more adaptive ways of thinking. Social skill development is also a primary focus, with children learning effective communication and conflict resolution strategies to foster positive peer relationships. In addition, parental involvement is an essential part of the program, as parents are trained to reinforce the skills taught in the program and to create a supportive environment at home. Finally, the program incorporates engaging activities such as games, role-playing, and storytelling, which allow children to learn about emotions and coping strategies in an enjoyable and interactive way.

The theoretical foundation of the Fun FRIENDS program is rooted in CBT, which is widely recognized for its effectiveness in treating anxiety and behavioral issues in children [41]. The program also draws on the social learning theory, which emphasizes the role of observational learning and reinforcement in the development of adaptive behaviors [42]. Considering these theoretical foundations and the proven success of the program in previous studies, it was selected as the intervention for our study.

The age range of 4–5 years is a crucial period for the development of social-emotional skills. During this time, children make substantial progress in emotional understanding and expression, establishing relationships with others, and developing self-regulation skills. This period is transitional, laying the foundation for developing key social-emotional competencies. Specifically, 4- to 5-year-olds experience rapid growth in emotional recognition, empathy, and self-regulation, while also developing an understanding of social rules and the perspectives of others. These skills play a significant role in later academic success, interpersonal relationships, and psychological adjustment.

The targeted age range of 4–5 years is particularly significant within the context of Japan's preschool system due to its alignment with key educational milestones. During these years, children in Japan begin their formal educational journey in kindergarten, a stage that is culturally important to their cognitive development and social and emotional skills. As children transition from home to a more structured environment, they are expected to acquire essential social competencies, such as cooperation, sharing, and emotional regulation, which are integral to Japan’s cultural emphasis on group harmony and societal conformity.

Takahashi et al. (2017) highlight that early childhood, especially the time between the ages of 4 and 5, is a crucial period for the development of social-emotional competencies [35]. These competencies lay the foundation for later academic success and emotional well-being. Meanwhile, Denham et al. [43] highlighted that 4-year-olds’ development of emotional regulation and social adaptation skills has lasting effects on their later academic performance and social relationships. Additionally, Zhou et al. [44] found that the development of social-emotional competencies is a crucial factor for success in school and learning. These findings suggest that focusing on 4–5-year-olds is critical as this period provides a foundation for later psychological adjustment and academic success [45].

Accordingly, early intervention at this stage can mitigate the challenges posed by cultural expectations, such as the pressure to conform and suppress individual emotions in favor of group cohesion. According to Oishi and Tanaka (2019), children who develop strong social-emotional skills early in life are better able to cope with these societal demands without compromising their emotional needs [46]. Along these lines, studies have shown that early SEL programs can reduce the likelihood that children will experience social anxiety or behavioral issues. For example, Kawai et al. (2021) found that interventions at the 4- to 5-year age range significantly improved social competence and emotional regulation, which are essential for children to thrive in group-centered educational settings [47]. By addressing these cultural challenges early on, SEL interventions can support children in developing the skills necessary for navigating both their educational environments and broader social contexts.

Recent studies have highlighted the importance of SEL programs in promoting psychological resilience and emotional regulation in young children [48, 49]. Among these, the Fun FRIENDS program has been widely adopted in Western educational systems, where it has demonstrated effectiveness in reducing anxiety and fostering social skills in early childhood [50]. However, there remains a notable gap in the literature regarding the application and impact of Fun FRIENDS in non-Western countries, particularly Japan. As noted above, Japanese educational settings present distinct cultural and pedagogical characteristics, such as a collectivist orientation and unique social expectations, that may influence the implementation and outcomes of SEL interventions [51]; accordingly, it is necessary to study the effectiveness of the program in Japan. This study addressed this gap by investigating the effectiveness of Fun FRIENDS within a Japanese preschool context, aiming to expand the cross-cultural validity of SEL programs and adapt them to different educational needs.

Verifying the effectiveness of Fun FRIENDS in Japan contributes to the broader field of early childhood education by highlighting potential cultural adaptations required for SEL programs. Insights from this study may inform educators and policymakers in Japan and similar settings, facilitating the design of culturally responsive SEL interventions that respect local practices while promoting universal social-emotional competencies. Given the increasing recognition of SEL’s role in academic and life success globally [52], this research addresses an urgent need for evidence-based SEL programs tailored to the unique sociocultural dynamics of Japanese early childhood education.

Notably, to align the program with the Japanese context, we made several adjustments. First, recognizing that Japanese culture places significant value on group harmony (wa) and collective well-being rather than on individual emotional expression, we modified certain activities to align with these cultural values. For example, the focus of some exercises was shifted from personal emotional identification to enhancing empathy and cooperation within a group context, encouraging children to consider the emotions of others in the group. Second, the language used in the program was carefully adjusted to reflect cultural sensitivities and nuances. In Japan, there are specific linguistic markers for expressing emotions that differ from those used in Western contexts. We ensured that the scenarios and dialogues used in the program resonated with Japanese children's understanding of emotions, particularly in relation to the common social situations they may face, such as those in school or family settings. Third, recognizing the importance of culturally appropriate teaching methods, we provided additional training for facilitators. This training not only emphasized the delivery of the program content but also the importance of understanding and responding to the emotional cues of children within the cultural framework. Facilitators were trained to be sensitive to non-verbal emotional cues, as emotional expression in Japan is often more subtle than in Western countries.

These modifications are consistent with findings from prior research that highlight the importance of adapting SEL interventions to cultural contexts to ensure their effectiveness. Greenberg et al. (2003) emphasize that SEL programs need to be tailored to the cultural and educational settings in which they are implemented [53]. Similarly, studies by Sumi et al. (2015) underline the importance of considering cultural values, such as the preference for indirect communication and emotional restraint in Japanese society, when designing SEL programs [54]. These adaptations were integral in making the Fun FRIENDS program both culturally relevant and effective for Japanese children. Ultimately, by making these cultural adaptations, we were able to address the challenges of SEL implementation in Japan and ensure that the Fun FRIENDS program met the emotional and social developmental needs of children in this context.

In Japan, there are few preventive interventions designed for young children that teach social skills, and while the effectiveness of the Fun FRIENDS program for elementary school students has been studied [55], its effectiveness for young children in the prevention of school maladjustment remains to be demonstrated. This study responded to this gap by introducing Fun FRIENDS into a Japanese kindergarten and measuring the social-emotional skills of the participants before and after the program to verify its effectiveness. We hypothesized that if social-emotional skills improved, problematic behaviors would decrease.

## Methods

### Participants

In 2022, we implemented the SEL program “Fun FRIENDS” on a class-by-class basis for 4–5-year-old children enrolled in kindergarten classes in Wakayama Prefecture, a suburban area of Japan. The intervention group consisted of two preschools that had an existing interest in SEL and expressed a desire to implement the Fun FRIENDS program. These facilities were selected based on their enthusiasm for fostering social-emotional skills.

In this study, participants were not randomly assigned to the intervention and control groups. Instead, the researchers worked closely with the participating kindergartens to ensure that the allocation process took into account the characteristics of the children to minimize any potential bias. The allocation was made at the facility level, with the aim of balancing the demographic variables across the intervention and control groups. This decision was made in collaboration with the kindergartens. The children’s characteristics, such as age, gender, and socio-economic status, were discussed to ensure that both groups were as comparable as possible.

While randomization is often considered the gold standard for group assignment, non-random allocation methods, such as the one employed in this study, have been shown to effectively minimize bias when randomization is not feasible. Research by Durlak et al. (2011) highlights that such carefully controlled, non-randomized designs can still provide valid results, particularly when selection bias is minimized through thoughtful allocation procedures [9].

The control group consisted of three preschools that agreed to implement an SEL program the following year. A total of 115 children from the two kindergartens participated in the Fun FRIENDS program, and 93 children divided into three classes did not participate in the program. In the intervention group, the entire class underwent the intervention at the facility with the approval of the facility representative. Children with language disorders or pervasive developmental disorders (intervention group: *n* = 11, control group: *n* = 4) were excluded from the statistical analysis. However, it is worth noting that these children were also offered the opportunity to participate in the intervention program. Disability assessment was based on information provided by both parents and teachers. Finally, 94 participants were included in the intervention group and 66 in the control group.

To ensure adequate statistical power for detecting meaningful effects, a power analysis was performed using G*Power software [56]. With 94 participants in the intervention group and an assumed medium effect size (Cohen’s d = 0.5), the calculated power of the study was approximately 0.80 at an alpha level of 0.05. This level of power is considered sufficient for detecting medium-sized effects in psychological and educational interventions [57]. Given that medium-sized effects are commonly observed in SEL programs, this level of power provides confidence that the findings are both reliable and meaningful.

The effect sizes for externalizing and internalizing behaviors in this study were 0.744 and 0.653, respectively, suggesting a moderate to large impact of the Fun FRIENDS program on children's psychological adjustment. To contextualize these findings, it is important to compare them with previous research on the Fun FRIENDS program and other SEL interventions.

In research conducted in Western countries, such as that by Barrett et al. (2011), the effect sizes for externalizing behaviors in children who participated in the Fun FRIENDS program ranged between 0.5 and 0.7, indicating moderate to large effects. Similarly, for internalizing behaviors, effect sizes ranged from 0.4 to 0.6 [58]. These findings are consistent with our study's effect sizes, suggesting that the Fun FRIENDS program has comparable efficacy across different cultural contexts.

However, our study's effect size for externalizing behaviors (0.744) is slightly higher than the effect sizes for externalizing behaviors typically reported in Western settings. This may reflect specific cultural factors in Japan that may amplify the impact of the program on externalizing behaviors. For example, as noted above, Japanese children often face heightened societal expectations regarding group conformity and behavior regulation, which may make them particularly receptive to interventions that promote social-emotional skills. Furthermore, studies in Japan have shown that children in this context may experience unique pressures related to social harmony and peer relationships, which could explain the more pronounced effect on externalizing behaviors [59]. While the effect size for internalizing behaviors (0.653) is also within the range observed in other studies, it is important to recall that the Japanese educational system places a strong emphasis on emotional regulation and social interaction from an early age. This cultural emphasis on emotional control may enhance the program's effectiveness in addressing internalizing behaviors, aligning with findings from previous SEL research conducted in non-Western contexts [60].

Thus, while the effect sizes observed in this study are consistent with the literature, the slightly higher effect size for externalizing behaviors in the Japanese context may suggest that cultural factors influence the outcomes of the Fun FRIENDS program, enhancing its impact in addressing behavior problems in early childhood.

Children with a diagnosis of language disorder or pervasive developmental disorder were excluded from the statistical analysis to ensure that the results were not confounded by developmental trajectories that differ significantly from typical patterns [61, 62]. This decision was based on the potential for these conditions to introduce variability unrelated to the intervention itself.

### Intervention

The Fun FRIENDS program aims to equip children with strategies to cope with anxiety and stress while building resilience and self-confidence to overcome difficulties. In this study, the program consisted of 10 sessions, each an hour long, conducted weekly. The Fun FRIENDS program addresses multiple domains of SEL by teaching children cognitive-behavioral strategies. In addition, the skills are delivered in a manner appropriate to the children’s developmental stage by utilizing play-based activities. In this study, the program was delivered by facilitators with expertise in this field, and multiple facilitators were employed to ensure the quality of the program. The facilitators received prescribed training before the start of the intervention. The control group received traditional instruction.

The Fun FRIENDS program was implemented with a focus on maintaining fidelity to the original model developed by Barrett et al. [63], with several key adjustments to suit the Japanese cultural context. Facilitator training was an essential component of this adaptation. All facilitators underwent the same rigorous training process as outlined in the original program but with an additional cultural sensitivity module. This ensured that facilitators were well-prepared to deliver the program in a culturally appropriate manner, aligning with research conducted by Fazel et al. [64], emphasizing the importance of culturally responsive training for program effectiveness.

To maintain the integrity of the program, fidelity was assessed using a checklist derived from the original measures described by Barrett et al. [63]. Facilitators were observed during sessions, and their reports were reviewed to ensure that the core elements of the program were delivered as intended. These fidelity checks, similar to those used in previous studies [64], allowed for continuous monitoring and adjustments throughout the implementation phase.

Specific adaptations were made to the program content taking into account the distinct cultural context of Japan. Examples and activities were modified to reflect Japanese societal values and communication styles, consistent with the recommendations of Elliott et al. [65], who argue that cultural adaptations are crucial for the success of international SEL programs.

To ensure the Fun FRIENDS program was culturally and educationally relevant for Japanese preschool children, several modifications were made to the curriculum and delivery approach. The original program, developed in Australia, emphasizes resilience-building strategies such as relaxation, emotional recognition, and problem-solving. While these core principles were retained, adaptations were made to align with Japanese cultural norms and educational practices.

First, the language and examples used in the program were adjusted to reflect common social situations encountered by Japanese children. For instance, scenarios involving group harmony and interpersonal relationships, which are culturally emphasized in Japan, were incorporated to enhance relatability. Studies have highlighted that collectivist cultures prioritize group cohesion and context-sensitive emotional understanding, making such adaptations crucial [66, 67].

Second, the delivery format was modified to fit the typical Japanese preschool schedule, which often includes structured group activities and strong teacher-led instruction. Activities promoting individual expression were balanced with group-based tasks to align with these practices while maintaining the program’s emphasis on fostering independence and self-confidence [68].

Additionally, the materials were visually adjusted to suit Japanese aesthetics, which often favor simplicity and cultural motifs. Feedback from preschool teachers and parents was integrated during the program's pilot phase to ensure appropriateness and acceptability. This participatory approach mirrors findings that culturally adapting interventions increases their effectiveness and engagement [69].

Lastly, training for facilitators emphasized the use of culturally appropriate communication strategies, such as indirect encouragement and non-verbal affirmation, which are more prevalent in Japanese educational settings [70]. Facilitators also received guidance on managing potential language nuances, particularly in teaching emotional vocabulary, an area where Japanese children may have less exposure compared to their Western counterparts [71].

These modifications aimed to preserve the program’s evidence-based components while ensuring cultural resonance. Preliminary feedback from participants and facilitators indicated that these adaptations enhanced engagement and comprehension, highlighting the importance of culturally sensitive intervention design in cross-cultural implementation.

In the control group, participants engaged in traditional instruction, which consisted of general classroom management strategies and basic social-emotional skills development activities. These activities included occasional teacher-directed discussions and informal role-playing exercises, but did not follow a structured or evidence-based curriculum as that used in the Fun FRIENDS program. Unlike the intervention group, which participated in a structured program targeting emotional regulation, resilience, and coping strategies through explicit teaching methods, the control group received more passive instruction focused on general social interaction skills [72, 73]. The traditional instruction was not organized into specific sessions nor did it involve the same level of interactivity and skill-building exercises that characterize the Fun FRIENDS program. As such, children in the two groups had fundamentally different experiences, with the intervention group benefiting from a more systematic and comprehensive approach to social-emotional skill development.

### Scale

#### Objective variable

In this study, children’s behavior was assessed using the Strengths and Problems Questionnaire (SDQ) [74–77]. The SDQ is a brief screening test to identify behavioral and emotional problems and prosocial behavior, consisting of 25 questions. We used the Japanese version, which has high reliability and validity. The SDQ consists of 20 of 25 items that assess behavioral and emotional problems, and these items are rated on a three-point Likert scale. Emotional and behavioral problems include conduct problems, hyperactivity and inattention, emotional symptoms, and peer relationship problems. Extroversion problems are determined by the total score of conduct problems, hyperactivity, and inattention. Introversion problems are determined by the total score of emotional symptoms and peer relationship problems. Higher scores indicate more emotional and behavioral problems.

The Japanese version of the Strengths and Difficulties Questionnaire (SDQ) has demonstrated strong psychometric properties, making it a reliable and valid tool for assessing emotional and behavioral problems in children. In a study conducted by Muris et al. [78], the SDQ showed good internal consistency (Cronbach’s α = 0.70–0.85) and test–retest reliability (r = 0.80). Furthermore, the validity of the SDQ in the Japanese population is supported by its ability to distinguish between clinical and non-clinical groups [79]. A more recent study by Kubo et al. [80] found that the factor structure of the SDQ remained stable across different age groups, further confirming its robustness in diverse populations. These findings underscore the suitability of the SDQ for use in this study, particularly within the Japanese preschool context.

#### Explanatory variables

We used the Fun FRIENDS program intervention as an explanatory variable.

#### Attributes

We collected parents’ self-reported information on their children’s family structure, household income, and education level.

#### Statistical analysis

First, we used chi-square tests to examine whether there were differences in the demographics of participants in the intervention and control groups at baseline. Second, we conducted analyses to examine whether there were differences in the delinquency scores of the intervention and control groups before and after the program. Furthermore, we conducted separate analyses for the intervention and control groups to examine whether there were changes in the delinquency scores after the program. All analyses were conducted using IBM SPSS Statistics Version 29.

To ensure data quality, we implemented several measures. First, data were entered independently by two researchers through a double data entry process. This method is widely used to minimize data entry errors [81], and we verified that both data entries were consistent. Furthermore, after data entry, we conducted error checks to identify and correct any missing or inconsistent values [82]. Additionally, we confirmed the reliability and validity of the measurement tools used, such as the Japanese version of the SDQ, based on previous studies that validated these tools for use in this population [83].

Specifically, in the data analysis section, we utilized paired-sample t-tests to compare the pre- and post-intervention scores, which allowed us to evaluate the direct effects of the intervention on the children's behavioral outcomes. In addition, we conducted multiple regression analyses to control for potential covariates, including age and gender, as these variables could have confounded the results. The results of these analyses are provided in Supplementary Tables 2 and 3.

Regarding the reliability and validity of the measures used to assess externalizing and internalizing behaviors, we focused on the Strengths and Difficulties Questionnaire (SDQ), which is widely used in research on child behavior. The SDQ has demonstrated strong internal consistency in our study, with Cronbach’s alpha values ranging from 0.71 to 0.88 across the different subscales. Furthermore, the test–retest reliability was high (r = 0.81), confirming the stability of the measure over time. These results are consistent with previous research on the SDQ's reliability and validity in the context of Japanese children [84]. These findings support the use of the SDQ as a robust tool for assessing externalizing and internalizing behaviors in this population.

### Ethical considerations

Study participants included all children in kindergarten classes aged 4–5 years, as the Fun FRIENDS program was a preschool education initiative. Regarding the assessment of children, the purpose and procedures of the study were explained to the children’s parents at the beginning of the study. The parents were informed that participation was voluntary. Before the children participated in the study, the parents signed written informed consent on their children’s behalf.

## Results

This study was not a randomized controlled trial (RCT), and as such, it did not involve typical randomization processes or group allocation. Therefore, instead of a traditional CONSORT-style flow diagram, we included a flow diagram outlining the participant recruitment process and their involvement in the follow-up and analysis stages. This approach, which enhances the transparency of our study’s reporting, was informed by previous research that emphasized the importance of adapting reporting guidelines for non-randomized studies to ensure clarity [85].

### Study participants’ attributes at T1

Table 1 presents the relevant demographic variables of the study participants at time T1, before the program implementation (baseline), and at time T2, after the implementation. Table 2 includes the baseline values for the outcome variables, with comparisons for the T1 measurements. These tables aim to provide a clear overview of the characteristics and initial conditions of both groups before the intervention commenced. Chi-square or t-tests were used to assess differences in the attributes of the intervention and control groups. None of the items exhibited significant differences. Further details of the statistical analysis, including confidence intervals for the main results, are provided in Supplementary Table 1.

**Table 1:** Study participants’ demographics at T1

		Intervention group (*n* = 94)		Control group (*n* = 66)		
	N	N (M)	% (SD)	N (M)	% (SD)	*p*-value
**Child’s sex**						
Male	81	51	62.96	30	37.04	0.273
Female	79	43	54.43	36	45.57	
**Child age**						
Average	4.76	4.72	0.33	4.82	0.33	0.074
**Family composition**						
Single-parent family	9	3	33.33	6	66.67	0.111
Two-parent family	151	91	60.26	60	39.74	
**Existence of siblings**						
0	32	18	56.3	14	43.8	0.748
1 +	128	76	59.4	52	40.6	
**Annual household income (million JPY)**						
< 4	31	17	54.84	14	45.16	0.796
≥ 4 – < 8	80	49	61.25	31	38.75	
≥ 8	30	17	56.67	13	43.33	
**Maternal educational level**						
Middle school or high school	60	36	60.00	24	40.00	0.167
Junior college or vocational school	55	34	61.82	21	38.18	
University or graduate school	37	16	43.24	21	56.76	
**Paternal educational level**						
Middle school or high school	53	32	60.38	21	39.62	0.913
Junior college or vocational school	31	18	58.06	13	41.94	
University or graduate school	62	35	56.45	27	43.55	

Chi-square tests and t-tests were performed

*Abbreviations: T1 *before program implementation (baseline), *N *number, *M *mean, *SD *standard deviation, *JPY *Japanese yen

**Table 2:** Comparison of problem behaviors from T1 to T2

	Intervention group			Control group		
	M	SD	*p*-value	M	SD	*p*-value
Externalizing problems						
T1	5.82	3.37	** < 0.001**	5.73	2.78	0.303
T2	4.11	3.51		5.44	2.75	
Internalizing problems						
T1	4.49	2.74	** < 0.001**	4.62	2.43	0.470
T2	3.11	2.38		4.45	2.55	

Paired-sample t-tests were conducted

*Abbreviations:* *T1 *before program implementation (baseline), *T2 *after program implementation, *M *mean, *SD *standard deviation

### Comparison of problem behaviors between the intervention and control groups

**Fig. 1:**
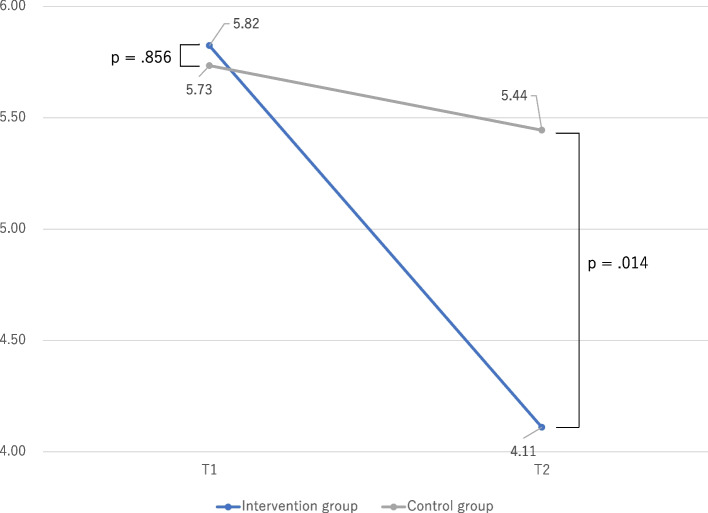
Comparison of problem behaviors between intervention and control groups: externalizing problems

**Fig. 2:**
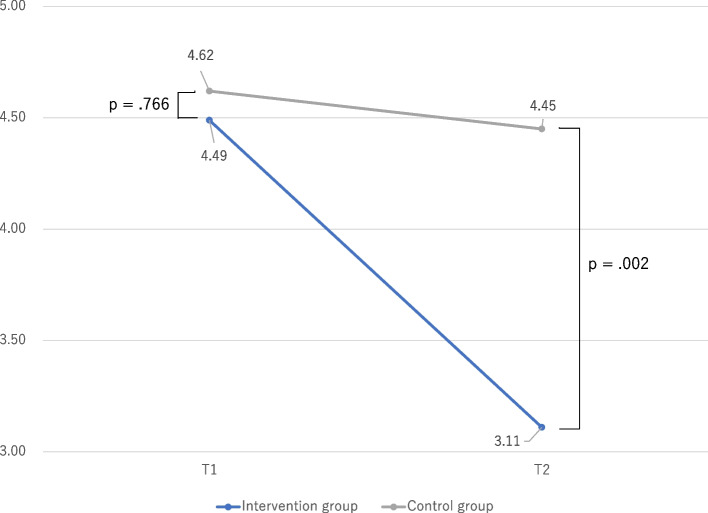
Comparison of problem behaviors between intervention and control groups: internalizing problems

We examined the differences in problem behaviors between the intervention and control groups using independent t-tests, as illustrated in Fig. 1 (externalizing problems) and Fig. 2 (internalizing problems). At T1 (before program implementation), the intervention group did not exhibit significant differences in problem behaviors related to externalizing and internalizing problems compared with the control group. However, at T2 (after program implementation), the intervention group demonstrated significantly lower problem behaviors in both externalizing and internalizing domains compared with the control group.

### Comparison of problem behaviors from T1 to T2

Table 2 examines the differences in problem behaviors at T1 and T2 using a corresponding t-test. In the intervention group, problem behaviors for externalizing and internalizing problems at T2 were significantly lower than at T1; however, in the control group, problem behaviors at T2 were not significantly different compared with T1 for both externalizing and internalizing problems. The effect size for externalizing behaviors in the target group was 0.744, while the effect size for internalizing behaviors was 0.653.

### Supplemental analysis

In addition, we conducted a subgroup analysis to evaluate whether the program remained effective across different baseline problem levels. To classify participants, we summed scores for externalizing and internalizing issues, designating individuals scoring 0–13 as the “normal group” and those scoring 14–40 as the “abnormal group” [74]. For each subgroup, paired-sample t-tests were applied to assess changes pre- and post-intervention.

Results demonstrated a significant reduction in problem risk in both the normal and abnormal subgroups within the intervention group, while no significant differences emerged in the control group before or after the program.

## Discussion

We evaluated the effectiveness of Fun FRIENDS, a universally designed preventive intervention program for preschool children. Comparisons of problem behaviors before and after the intervention revealed a significant decrease in extroverted and introverted problem behaviors in the intervention group compared to the control group (*p* < 0.05). We found that the Fun FRIENDS program may have improved social and emotional skills, resulting in the decrease in extroverted and introverted problem behaviors. However, statistical significance alone does not fully reflect practical relevance. For instance, when we evaluated the effect size using Cohen’s d, the difference between the intervention and control groups was more moderate (the effect size for externalizing behaviors in the target group was 0.744, while the effect size for internalizing behaviors was 0.653), suggesting that the effect may be of practical significance. Effect size serves as an important indicator, going beyond statistical significance to demonstrate how interventions impact real-world educational or social contexts [86, 87]. Therefore, the results show that implementing the Fun FRIENDS program leads to significant differences in problem behavior, suggesting that the program may have meaningful practical implications.

The Fun FRIENDS program is designed to promote social-emotional skills, which can significantly impact the reduction of problem behaviors in children. Several mechanisms may account for the observed improvements in behavior, each corresponding to specific components of the intervention.

One potential mechanism is cognitive restructuring and problem-solving. The program emphasizes helping children identify and challenge negative or unhelpful thoughts, which is crucial in reducing externalizing behaviors such as aggression or defiance. By teaching children to reframe their thoughts and adopt more adaptive, solution-focused strategies, the program encourages positive thinking patterns. Research indicates that cognitive restructuring can help children manage their emotions and reactions, leading to a decrease in problem behaviors [88]. This component of the program directly targets the reduction of reactive behaviors by promoting more balanced perspectives and effective coping strategies.

Another important mechanism is the development of emotion regulation skills. The Fun FRIENDS program teaches children to recognize, understand, and regulate their emotions in socially appropriate ways. Studies have shown that improving emotional regulation can significantly reduce both internalizing and externalizing problem behaviors [89]. By enhancing children’s ability to manage emotional responses to challenging situations, the program helps prevent behaviors such as aggression, impulsivity, or anxiety. This component is vital for children’s overall psychological adjustment, as it equips them with the tools to navigate emotional challenges effectively.

Social skills training also plays a crucial role in reducing problem behaviors. The program targets the development of interpersonal skills such as sharing, making friends, and cooperating with others. Research suggests that children who struggle with social skills are at greater risk of problem behaviors, including peer rejection, aggression, and school difficulties [90]. By improving social competence, the program helps children engage in more positive peer interactions, which in turn reduces externalizing behaviors like aggression and internalizing behaviors such as withdrawal or social anxiety.

Finally, the program fosters increased self-esteem and teaches coping skills. By boosting self-esteem and providing children with strategies to cope with stress, frustration, and disappointment, the program addresses behaviors associated with low self-worth or poor stress management. Children with higher self-esteem and better coping abilities are less likely to engage in both externalizing and internalizing problem behaviors [91]. Through these components, the Fun FRIENDS program helps children build resilience and emotional strength, further reducing problem behaviors.

In summary, the Fun FRIENDS program may reduce problem behaviors through a combination of mechanisms: cognitive restructuring, emotion regulation, social skills training, and increased self-esteem. These mechanisms are directly linked to the program’s core components, and research supports their role in promoting positive behavioral outcomes.

Social adjustment is greatly influenced by childhood behavior problems throughout the lifespan [92–94]. The Fun FRIENDS program teaches children cognitive-behavioral strategies that address multiple domains of SEL by cultivating social competence. The program focuses on reducing negative aspects and promoting positive protective factors. It aims to improve children’s understanding and ability to control their own emotions to acquire developmentally appropriate skills and develop self-regulation abilities.

The intervention group did not have lower problem behavior scores than the control group after program implementation. This is because the intervention group had higher scores on extraversion and introversion problems than the control group before the program began. The intervention group may have already had more problem behaviors, and therefore the effect may have been large and overestimated. Alternatively, the lack of significant results on the difficulties subscale of the SDQ may have been due to floor or basement effects, leading to underestimation. This issue requires further investigation. For example, future studies could align risk for problem behaviors at baseline between groups. Because we found a reduction in risk for problem behaviors, we could expand the knowledge gained from the relatively small number of Fun FRIENDS effects examined in this study [95]. Overall, our study provides evidence of the effectiveness of the Fun FRIENDS program as a universal school-based preventive intervention. Because children who attend kindergarten spend most of their day there, it serves as a common entry point for interventions aimed at a large number of children. In addition, the kindergarten environment provides a desirable approach to identify children in need of support and provide services appropriate for them. Therefore, kindergartens are an ideal place to implement preventive intervention programs aimed at promoting social-emotional competence in early childhood. A universal approach is one that is applied to the entire group or classroom. The Fun FRIENDS program teaches children cognitive-behavioral strategies that address multiple domains of SEL and transfers skills in a developmentally appropriate manner, utilizing play-based activities.

In this study, we evaluated the Fun FRIENDS program’s effectiveness in a Japanese preschool context. Our findings align with previous studies demonstrating the program’s positive impact on children’s social-emotional skills and reduction in problem behaviors. For instance, a study by Barrett et al. [67] found that Fun FRIENDS significantly improved social and emotional functioning in Australian children. Similarly, our study showed improvements in children’s emotional regulation and peer relationships. However, there are notable differences between our results and those of studies conducted in Western contexts.

While Barrett et al. [96] reported significant reductions in anxiety symptoms, our study did not observe a comparable effect on anxiety, possibly due to cultural differences in how anxiety is expressed and addressed in Japanese children. In Japan, social and emotional issues may be more likely to manifest in behavioral forms rather than verbal expressions of anxiety, which could explain the differing outcomes.

Another study by Lang et al. [97] conducted in the UK also found significant improvements in social skills and a reduction in externalizing behaviors following the Fun FRIENDS program. Our study similarly found improvements in externalizing behaviors, though the magnitude of change was smaller. This discrepancy could be explained by differences in baseline levels of behavioral problems in the samples used. Furthermore, the baseline scores for externalizing behaviors were lower, suggesting a ceiling effect that may have limited the potential for improvement.

These differences may also be attributed to the cultural context of Japan, where the importance of group harmony and emotional restraint might affect how children respond to SEL programs. Previous research has highlighted the role of cultural values in shaping the effectiveness of psychological interventions [98], and this is particularly relevant in the context of SEL programs.

The findings of this study have significant implications for early childhood education policy and practice in Japan. In particular, the observed effectiveness of the Fun FRIENDS program suggests that enhancing social-emotional skills can contribute to long-term psychological well-being and behavioral adjustment. Implementing such programs in educational settings could support children’s self-efficacy and interpersonal skills, which have been shown to influence future academic success and social adaptation [9, 99]. However, despite these promising benefits, there are several barriers to the implementation of these programs in Japan.

First, cultural factors may present a significant obstacle. In Japan, there may be limited understanding and support for programs that promote self-expression and self-esteem development [100], leading to cautious progress in implementing such programs. Additionally, insufficient training for educators within early childhood facilities poses a challenge to effective program delivery [101]. Providing structured support and training for early childhood educators could reduce barriers to program implementation.

At a policy level, resource allocation in the education system is crucial to making SEL programs an integral part of early childhood education. Considering the limited personnel and time resources in the current system, support from government and local education authorities is essential. Specifically, increased budget allocation and legal backing from the government to support SEL initiatives could enable sustainable implementation in educational settings [102].

To overcome these barriers, programs should be adapted to align with cultural expectations, promoting self-efficacy and self-esteem while respecting Japan’s value of cooperation in educational contexts [103]. Additionally, providing ongoing professional development for teachers and caregivers would enhance their understanding of the program content and their ability to deliver it effectively. Finally, policymakers should ensure that educational facilities have the necessary resources and continue to promote the importance of social-emotional skill development in early childhood education [104]. By addressing these needs, the Fun FRIENDS program can achieve sustainable impact and widespread implementation, supporting the healthy development of young children in Japan.

The Fun FRIENDS program, which focuses on promoting SEL in early childhood, has shown promising outcomes in improving children’s psychological well-being and reducing problem behaviors. As we consider the implementation of the program on a larger scale, it is crucial to examine its potential cost-effectiveness, considering both the short-term benefits (e.g., reduced emotional and behavioral problems in children) and long-term advantages (e.g., improved academic performance and better mental health outcomes in later life).

In the short term, the costs associated with implementing Fun FRIENDS, such as facilitator training, program materials, and time spent on program delivery, may be significant. However, these initial investments are likely to result in immediate benefits, including improvements in children’s emotional regulation, social skills, and reductions in behavioral problems. Evidence suggests that early interventions that promote emotional and social competence can reduce the need for more intensive and expensive treatments later on [105]. In fact, implementing programs such as Fun FRIENDS in preschool settings may prevent the escalation of mental health issues, which could ultimately save on healthcare and educational costs [106].

The long-term benefits of the Fun FRIENDS program are also important. Early intervention programs that target SEL skills in young children have been associated with long-term positive outcomes, including enhanced academic achievement, improved mental health, and reduced involvement in criminal behavior [107]. These long-term benefits could offset the initial implementation costs, as children who develop strong emotional and social skills are more likely to experience greater success in education and life, leading to reduced societal costs in terms of healthcare, social services, and lost productivity [108]. Furthermore, the cost-effectiveness of implementing Fun FRIENDS on a larger scale may increase as the program reaches more children, thus reducing per-child implementation costs.

Several studies have highlighted the cost-effectiveness of SEL programs. Durlak et al. [78] conducted a meta-analysis showing that SEL programs not only yield significant benefits in terms of improved social-emotional skills but also result in a positive return on investment by reducing later mental health costs and improving academic performance. Similarly, Barton et al. [105] emphasized the economic advantages of investing in early intervention programs that foster social-emotional competence, noting that the benefits of reduced healthcare and educational expenditures over time are substantial.

Recent studies have demonstrated that the benefits of SEL interventions are not limited to the preschool years, but can extend into early school years, contributing to sustained improvements in social, emotional, and academic outcomes. For instance, Durlak et al. [9] found that the long-term gains in social skills, behavior, and academic achievement persisted beyond one year of follow-up. Furthermore, Jones et al. [109] reported that preschool children who received SEL interventions experienced continued academic and behavioral benefits throughout the early elementary grades, suggesting that interventions can provide children with a foundation for future success. These findings align with the objectives of the Fun FRIENDS program, which aims to promote emotional regulation, social competence, and resilience among young children, with the potential for these benefits to endure as children progress through school.

To scale the Fun FRIENDS program across various preschool settings, it is essential to provide comprehensive training for facilitators and adapt the program to different resource levels. Facilitator training should begin with an intensive professional development program that covers both the theoretical underpinnings of the program and practical strategies for its implementation. According to Domitrovich et al. [110], effective training must be hands-on, with opportunities for facilitators to practice delivery and receive feedback to build confidence and competence. Facilitators should also be trained in managing diverse classroom environments and addressing challenges such as large class sizes or varying levels of student engagement.

Moreover, the resources used in the program should be adapted to suit the needs of each preschool setting. This might involve modifying activities to fit the time constraints of the preschool day or adjusting materials to reflect local cultural contexts. Durlak et al. [49] highlighted the importance of tailoring program content and materials to the specific characteristics of the school, which increases the likelihood of successful implementation and sustained effects.

Ongoing support and supervision for facilitators are also critical to ensure fidelity to the program as it is scaled. Regular check-ins, coaching, and refresher training sessions are necessary to address implementation challenges and maintain the quality of the program over time. These strategies are aligned with the findings reported by Durlak et al. [49], which emphasized the significance of continuous professional development in maintaining program effectiveness when scaled.

Regarding the implications of our findings for the field of early childhood social-emotional development, our study aligns with a growing body of research that emphasizes the importance of social-emotional skills in early childhood as foundational for later academic and social success [72, 111]. The results of our study underscore the potential of targeted interventions, such as the Fun FRIENDS program, to foster these skills and contribute to positive psychological outcomes in young children.

Considering the increasing emphasis on early intervention programs, particularly in promoting social-emotional development [112], the scalability of such programs is of critical importance for policymakers and educators. In this context, our findings contribute valuable insights into how such programs can be effectively implemented, particularly within different cultural contexts like Japan [113].

We provided a comparison of our study’s findings with similar interventions, emphasizing how the Fun FRIENDS program contributes uniquely to the existing literature. While other studies have explored the effectiveness of SEL programs, our study fills a gap by verifying the program’s efficacy in the Japanese context, thus adding to the international body of knowledge on early childhood SEL interventions.

Overall, our results highlight the importance of implementing programs that promote social-emotional skills and resilience from an early age in terms of preventing behavioral problems [114, 115].

### Limitations and future prospects

Some limitations of this study should be acknowledged. First, follow-up was limited. Childhood interventions require long-term follow-up to capture their potential effects and avoid underestimating the effectiveness of the program [116]. In addition, baseline levels of problem behavior scores were significantly different between the intervention and control groups in this study. Future research designs incorporating cluster randomization or similar methods are needed. Furthermore, the assessment of problem behavior was based on parental reports. Future studies should incorporate other assessment methods, such as child or third-party observation reports (although practical constraints make it difficult to introduce other assessment methods of treatment outcomes in community-based mental health settings). Moreover, the sampling strategy used in this study had a potential selection bias because the participating facilities were already interested in developing social-emotional skills, raising the possibility of overestimating the effects of the SEL program, as previously described [117–119]. In this regard, future research should examine the generalizability of the results by conducting a sample survey of a more diverse range of facilities. In addition, the exclusion of children with language disorders and pervasive developmental disorders may limit the external validity of the study [62, 120]. Future research should explore the effectiveness of the intervention in these populations to better understand its potential benefits for a broader range of children. The potential for selection bias may limit the generalizability of our results. As the preschools in the intervention group were those already interested in SEL, there is a possibility that the participants’ pre-existing interest influenced the observed outcomes. Such bias may lead to overestimating the program’s effectiveness, as participants were already motivated to improve their social-emotional skills. Similar challenges related to selection bias have been documented in studies involving self-selected samples in educational interventions [121]; the use of self-reported measures from caregivers and teachers introduces potential measurement bias. Informants’ perceptions of children’s behaviors may be influenced by subjective expectations or social desirability, potentially skewing the data. This limitation has been noted in educational and psychological research, as informant bias can compromise the validity of observed outcomes [122]. Future research should consider incorporating objective measures, such as observational data, to complement the self-report instruments used here. Lastly, while this study’s findings contribute to understanding the effectiveness of the Fun FRIENDS program in Japan, caution should be exercised when generalizing these results beyond similar cultural or educational contexts. Differences in educational practices and cultural norms may impact the program’s applicability in other settings, as shown in cross-cultural studies of SEL interventions [9]. Future research should seek to replicate these findings across more diverse populations to strengthen external validity. In this study, we did not use cluster randomization, which is a common strategy for minimizing baseline discrepancies between intervention and control groups in RCTs. Instead, participants were allocated to the intervention or control group based on the facilities’ interest in participating. This method of allocation could have introduced selection bias [123], as facilities already invested in SEL might differ systematically from those not as invested. While this approach did not involve random allocation at the group level, we took steps to address potential baseline discrepancies between the intervention and control groups. Specifically, we included relevant baseline covariates (such as age, sex, and pre-intervention psychological adjustment scores) in the statistical analyses to control for potential confounding variables and minimize baseline differences. This approach allowed us to account for initial differences between the groups and more accurately assess the effect of the intervention. The current study primarily relied on parent-reported data and child assessments to evaluate the effectiveness of the intervention. However, incorporating teacher feedback and direct observations would provide a more comprehensive and holistic evaluation of the program’s impact. Teachers are integral in monitoring children’s behavior and social-emotional development in the classroom, and their insights could offer a complementary perspective to parent reports. Direct observations in particular could allow for more objective assessments of children’s interactions, emotional regulation, and social behaviors in real-world settings. In future studies, it would be valuable to include teacher feedback and direct observations as additional measures to assess the effectiveness of social-emotional interventions. This approach has been advocated in several studies, where teachers’ perspectives and observations have been shown to provide valuable insights into the dynamics of children’s social development that may not be fully captured through parent-reports alone [124, 125]. Incorporating these methods could enhance the robustness of the findings and offer a more well-rounded evaluation of the intervention’s outcomes. The lack of teacher input in the current study represents a further limitation that we plan to address in future research. Incorporating teacher observations and feedback will help to ensure a more complete understanding of the intervention’s impact on children across various settings, ultimately contributing to more effective and targeted interventions for early childhood education. Finally, this intervention was only targeted at children. To ensure better practice at home, full-scale interventions for parents are needed in conjunction with preschool education.

## Conclusions

We evaluated the effectiveness of Fun FRIENDS, a universal preventive intervention program for preschool children. We hypothesized that a reduction in the risk of problem behaviors would be observed after the intervention was completed. This hypothesis was supported, and a comparison of the pre-intervention and immediate post-intervention results showed that the intervention group had reduced problems with extroversion and introversion compared to the control group. Our results suggest a promising direction for the development of preventive interventions for young children. Although this program is unique as a child-centered intervention program and further research is needed, direct interventions aimed at children seem to be an effective strategy for improving social competence. In addition, this study was conducted in a general early childhood education setting, and the program was practical. These results can be used to help disseminate the Fun FRIENDS program in future preventive interventions.

Based on the positive outcomes observed in this study, we recommend that future research include long-term longitudinal studies to investigate the long-term effects of the Fun FRIENDS program. These studies could assess whether the improvements in social-emotional skills and reductions in problem behaviors observed immediately after the intervention are sustained over time and how these effects may influence children’s development in areas such as academic achievement, peer relationships, and mental health. Longitudinal investigations could also reveal delayed effects of the program, such as improvements in emotional regulation or social interactions that manifest months or even years after the program’s completion. Additionally, examining the potential for differential effects based on baseline characteristics, such as age, sex, and socio-economic status, could provide valuable insights into how the program may be further tailored to meet the needs of diverse populations.

Another important avenue for future research involves examining the mechanisms through which the program produces its effects [126]. Future studies should focus on identifying the components of the intervention—such as its social skills training, cognitive-behavioral strategies, or emotional regulation techniques—that mainly contribute to the observed outcomes. This knowledge would help optimize the program and ensure that its benefits are maximized in future implementations. Furthermore, it would be beneficial to explore the feasibility and effectiveness of implementing the Fun FRIENDS program in various cultural and socio-economic contexts to determine its generalizability and identify potential barriers to its widespread adoption and sustainability.

## Supplementary Information

Supplementary Material 1.

## Data Availability

The datasets generated and/or analyzed during this study are available from the corresponding author upon reasonable request.

## Abbreviations

CASEL: Collaborative for Academic, Social, and Emotional Learning
SD: Standard deviation
SDQ: Strengths and Difficulties Questionnaire
SEL: Social and emotional learning
