# Supplementary material for: Enhancing social-emotional skills in early childhood: intervention study on the effectiveness of social and emotional learning
Source: BMC Psychol. 2024 Dec 18;12:761. doi: 10.1186/s40359-024-02280-w (PMC11657640; doi:10.1186/s40359-024-02280-w)
Supplement: Supplementary file 1 — Supplementary Material 1. [file 40359_2024_2280_MOESM1_ESM.docx]

**Supplementary Table 1**

*Comparison of Changes in Problem Behaviors from T1 to T2*

|  | Intervention group | | | | | Control group | | | | |
| --- | --- | --- | --- | --- | --- | --- | --- | --- | --- | --- |
|  | M | SD | 95% CI | | p-value | M | SD | 95% CI | | p-value |
| Externalizing problems |  |  |  |  |  |  |  |  |  |  |
| T1-T2 | 1.700 | 2.125 | 1.227 | 2.173 | **<0.001** | 0.258 | 1.958 | -0.239 | 0.755 | 0.303 |
| Internalizing problems |  |  |  |  |  |  |  |  |  |  |
| T1-T2 | 1.466 | 2.361 | 0.947 | 1.985 | **<0.001** | 0.205 | 2.201 | -0.359 | 0.769 | 0.470 |

Abbreviations: T1, before program implementation (baseline); T2, after program implementation; mean (M); standard deviation (SD); 95% Confidence Interval (95% CI). Paired-sample t-tests were conducted.

**Supplementary Table 2**

*Relationship between the Implementation of SEL and Changes in Externalization Problems*

|  | *B* | *SE* | *β* | *P* | *Adjusted R^2^* | *VIF* |
| --- | --- | --- | --- | --- | --- | --- |
| SEL implementation | -1.206 | 0.372 | -0.296 | 0.002 | 0.124 | 1.046 |
|  |  |  |  |  |  |  |
| Child’s sex | -0.441 | 0.364 | -0.109 | 0.228 |  | 1.021 |
| Family composition | 0.158 | 1.417 | 0.010 | 0.911 |  | 1.035 |
| Existence of siblings | 0.031 | 0.460 | 0.006 | 0.946 |  | 1.061 |
| Annual household income | -0.260 | 0.305 | -0.080 | 0.396 |  | 1.113 |
| Maternal educational level | 0.296 | 0.265 | 0.114 | 0.266 |  | 1.307 |
| Paternal educational level | 0.170 | 0.224 | 0.075 | 0.449 |  | 1.229 |

Abbreviations: unstandardized coefficient (B); standard error (SE); standardized coefficient (β), social and emotional learning implementation (SEL implementation); Intervention group = 1, Control group = 0, Multiple regression analysis was conducted.

**Supplementary Table 3**

*Relationship between the Implementation of SEL and Changes in Internalization Problems*

|  | *B* | *SE* | *β* | *P* | *Adjusted R^2^* | *VIF* |
| --- | --- | --- | --- | --- | --- | --- |
| SEL implementation | -1.193 | 0.410 | -0.254 | 0.004 | 0.196 | 1.059 |
|  |  |  |  |  |  |  |
| Child’s sex | 0.315 | 0.400 | 0.068 | 0.433 |  | 1.035 |
| Family composition | -2.449 | 1.555 | -0.136 | 0.118 |  | 1.037 |
| Existence of siblings | -0.888 | 0.496 | -0.157 | 0.076 |  | 1.058 |
| Annual household income | 0.380 | 0.326 | 0.104 | 0.247 |  | 1.106 |
| Maternal educational level | -0.067 | 0.292 | -0.022 | 0.818 |  | 1.309 |
| Paternal educational level | 0.498 | 0.246 | 0.190 | 0.046 |  | 1.225 |

Abbreviations: unstandardized coefficient (B); standard error (SE); standardized coefficient (β), social and emotional learning implementation (SEL implementation); Intervention group = 1, Control group = 0, Multiple regression analysis was conducted.

**Supplementary Table 4**

*Comparison of Problem Behaviors from T1 to T2 in the Normal Group*

|  | Intervention group (n=82) | | | Control group (n=57) | | |
| --- | --- | --- | --- | --- | --- | --- |
|  | M | SD | p-value | M | SD | p-value |
| T1 | 8.899 | 3.723 | **<0.001** | 9.055 | 3.223 | 0.978 |
| T2 | 5.886 | 3.946 |  | 9.069 | 3.905 |  |

Abbreviations: T1, before program implementation (baseline); T2, after program implementation; mean (M); standard deviation (SD). Paired-sample t-tests were conducted.

**Supplementary Table 5**

*Comparison of Problem Behaviors from T1 to T2 in the Abnormal Group*

|  | Intervention group (n=12) | | | Control group (n=9) | | |
| --- | --- | --- | --- | --- | --- | --- |
|  | M | SD | p-value | M | SD | p-value |
| T1 | 18.222 | 1.563 | **0.014** | 19.833 | 3.563 | 0.134 |
| T2 | 15.056 | 4.693 |  | 15.173 | 5.159 |  |

Abbreviations: T1, before program implementation (baseline); T2, after program implementation; mean (M); standard deviation (SD). Paired-sample t-tests were conducted.
